# Secure and Scalable mHealth Data Management Using Blockchain Combined With Client Hashchain: System Design and Validation

**DOI:** 10.2196/13385

**Published:** 2019-05-16

**Authors:** Tomomitsu Motohashi, Tomonobu Hirano, Kosuke Okumura, Makiko Kashiyama, Daisuke Ichikawa, Taro Ueno

**Affiliations:** 1 SUSMED, Inc Tokyo Japan

**Keywords:** mobile health, electronic health records, blockchain, client hashchain, clinical trial

## Abstract

**Background:**

Blockchain is emerging as an innovative technology for secure data management in many areas, including medical practice. A distributed blockchain network is tolerant against network fault, and the registered data are resistant to tampering and revision. The technology has a high affinity with digital medicine like mobile health (mHealth) and provides reliability to the medical data without labor-intensive third-party contributions. On the other hand, the reliability of the medical data is not insured before registration to the blockchain network. Furthermore, there are issues with regard to how the clients' mobile devices should be dealt with and authenticated in the blockchain network in order to avoid impersonation.

**Objective:**

The aim of the study was to design and validate an mHealth system that enables the compatibility of the security and scalability of the medical data using blockchain technology.

**Methods:**

We designed an mHealth system that sends medical data to the blockchain network via relay servers. The architecture provides scalability and convenience of operation of the system. In order to ensure the reliability of the data from clients' mobile devices, hash values with chain structure (client hashchain) were calculated in the clients' devices and the results were registered on the blockchain network.

**Results:**

The system was applied and deployed in mHealth for insomnia treatment. Clinical trials for mHealth were conducted with insomnia patients. Medical data of the recruited patients were successfully registered with the blockchain network via relay servers along with the hashchain calculated on the clients' mobile devices. The correctness of the data was validated by identifying illegal data, which were made by simulating fraudulent access.

**Conclusions:**

Our proposed mHealth system, blockchain combined with client hashchain, ensures compatibility of security and scalability in the data management of mHealth medical practice.

**Trial Registration:**

UMIN Clinical Trials Registry UMIN000032951; https://upload.umin.ac.jp/cgi-open- bin/ctr_e/ctr_view.cgi?recptno=R000037564 (Archived by WebCite at http://www.webcitation.org/78HP5iFIw)

## Introduction

Digital medicine, including the use of mHealth apps and internet of things (IoT) devices, has become popular in the everyday practice of medicine [1]. It has the potential to promote improved patient health outcomes, support care coordination, and improve communication with lower costs. While digital medicine has the potential for better practices to patients, we need to consider the security issues. Data tampering and impersonation are important security risks for digital medicine and clinical trials. Decision making in medical practice should be based on precise patient information. Data reliability is compromised if data tampering and impersonation are used to attack the system. External cyberattacks, including ransomware attacks, which result in compromised medical records, are huge threats against the health care sector [2,3]. Data breaches can lead to privacy violations, embarrassment, and social stigma, as well as to fraud and medical identity theft.

In addition to cybersecurity, data governance and authenticity are also important issues in the health care sector, especially in data management in clinical trials [4,5]. Recently, Web-based clinical trials have been conducted to streamline and improve the convenience of clinical trial participation [6]. Since the results of the clinical trials are the basis of the approval of medicine or medical devices by regulatory agencies, the reliability and transparency of the data obtained by the clinical trial must be maintained [7]. However, there are reports that 17% of clinical drug trials were fabricated [8-10]. The ability to easily trace data back to the original source is indispensable.

Blockchain technology has recently garnered attention as a means for transferring data between participating parties based on a “distributed ledger” model that affords a fully transparent and immutable record of data transactions [11]. A blockchain consists of a continuously growing list of transactional records organized into blocks that are replicated on the nodes of a peer-to-peer network. Valid transactions stored in a blockchain are digitally signed and timestamped by their sender, providing cryptographically irrefutable evidence of both provenance and existence of a record at a given point in time. The technology provides a verifiable and tamper-proof history of the data in the blockchain network. Bitcoin is the first implementation of blockchain as a digital asset in widespread use [12]. It eliminates the need for trusted third parties in financial transactions by providing a secure and verifiable history for every transaction in the system.

Beyond digital currency, researchers have started to focus on using blockchain technology for building cryptographic proof in many areas including medical sectors [13]. Blockchain has already been proposed for use in various health care settings, with potential applications in health supply chain management [14-16], insurance claims processing [17-19], medical record management [20,21], and data management in clinical trials [22-26]. Drug counterfeiting is a global problem with significant risks to consumers and the general public. Blockchain has the potential for tracking and tracing drug products and reagents, and counterfeit detection through information verification of supply chain participants. Blockchain technology can also be applied to managing insurance claim policies by the insurance providers and the patients. It can provide authorized access of data to researchers to analyze diseases. Blockchain-based models for electronic medical records have been proposed to enhance ownership of their medical data and data sharing between platforms [17,27,28]. Since the blockchain can be used to establish a permanent record agreed on by all participating parties, it has the potential to mitigate some of the threats to data validity, so that some researchers have proposed to support or even replace the traditional data infrastructure used in clinical trials with blockchain systems [22,23,29,30]. Our previous study also demonstrated an mHealth system for insomnia using a mobile phone app together with a blockchain storage platform and evaluated resistance against tampering of the data collected with mobile phones [24].

Although medical data registered in a blockchain network have proved tamper-resistant, the vulnerability of the medical data lies before registration to the blockchain network. Impersonation of client devices or fabrication of data outside of the blockchain network can impair the reliability of the medical data. In addition, if the blockchain network is open to the Internet, the network is vulnerable to attack and to the theft of medical records. Client devices, such as mobile phones used by patients, should not be dealt with as nodes of the blockchain network in order to preserve the confidentiality of the personal medical data and to reduce the operational cost for the management of the private key. The trade-off must be recognized and overcome by technological improvements.

This study aims to describe and validate an mHealth system using a client management architecture along with a blockchain network. To overcome the remaining problems described above, we designed the whole system with relay servers, which connect client devices to the blockchain network. In order to ensure reliability and to prevent impersonation or fabrication, a hashchain was calculated in client devices and sent to the blockchain network along with the medical data. We further verified the proposed system in the actual clinical trial of mHealth for insomnia patients and evaluated the resistance to various fraud attacks.

## Methods

### Clinical Trial and mHealth Records

The proposed system was applied and deployed into the mHealth app, which treats chronic insomnia based on cognitive behavioral therapy for insomnia (CBTi) [31]. After a favorable formal review by the Japanese Pharmaceuticals and Medical Devices Agency, research on the mHealth app was conducted by the digital therapeutics company, SUSMED, Inc. (Tokyo, Japan). Informed consent was obtained from the patients for publication of this study. The study has received ethical approval from the Ethics Committee and registered to clinical trial registry (UMIN000032951). All the methods were performed in accordance with the relevant guidelines and regulations.

The mHealth records collected from patients were divided into subjective and objective data. The subjective data, which include clinical indicators, sleep status, and the review of daytime activities, were collected through a self-administered questionnaire. The objective data, which include the results of the Psychomotor Vigilance Test [32], were evaluated by measuring the touch response using the function of mobile phones. For clinical indicators, Athens Insomnia Scale [33], Epworth Sleepiness Scale [34], and Quick Inventory of Depressive Symptomatology [35] were collected. For sleep status, time to go to bed, time to fall asleep, time to wake up, and time to get out of bed were recorded. Along with the medical information, timestamps of the app operation were collected. These clinical indicators were collected using a mobile phone app. All data were stored in the JavaScript Object Notation (JSON) format in the database.

### mHealth Data Transfer Via Relay Servers to Blockchain Network

The collected data from the patients’ devices were sent to the blockchain network via relay servers. We used three relay servers, and the app randomly selected two relay servers to send the data after the authentication of the client device. By deploying the relay proxy and setting the blockchain software development kits (SDKs) to write-only mode, the relay server sent the received data to the blockchain network. The authentication of the relay server was conducted with a single common authentication server. By configuring the Internet Protocol (IP) address restriction to the listed relay servers, the blockchain network, which contains the medical data, was protected against external attack. The blockchain network was made up of three organizations that contain two validating peers. Each account for nodes of the blockchain network and the relay servers were managed by independent departments in SUSMED, Inc. The strictness of the governance of the data management can be adjusted by individually managing the accounts of different stakeholders, such as pharmaceutical companies, contract research organizations, and regulatory agencies.

### mHealth Data Registration in the Blockchain Network

We used Hyperledger Fabric v1.0 to operate the blockchain network because Hyperledger is an open-source blockchain platform and has become widely used [36,37].

The blockchain network was administered by a collection of organizations. Each organization had multiple nodes. In this study, the network had three organizations and each organization had two nodes. As the state database, CouchDB was used to store the JSON document [38].

The nodes executed an installed chaincode and returned hash values generated from the execution result. The secure hash algorithm SHA-256 was used to compute the hash values encoded into the blocks of the blockchain [39]. To execute the transaction, each node followed the consensus algorithm, which was called endorsement policy [11], although the previous version of Hyperledger Fabric used Practical Byzantine Fault Tolerance as a consensus algorithm [40,41].

The endorsement policy was set in units of organizations, and the flexible set was available according to the needs of the app. Each organization issued one signature. The node that validated the transactions in each organization was called the endorser. In this study, the validation of each transaction required more than two signatures from three organizations.

Under the endorsement policy, transactions were validated and accepted in the following processes:

1. Proposal: The transaction was sent from the client app to the endorsers in each organization.
2. Endorse: Each endorser verified that (1) the transaction proposal was well formed, (2) it had not been already submitted in the past, (3) the signature was valid, and (4) the client was properly authorized to perform the proposed operation, which was described in the chaincode. If the transaction was validated, chaincode was executed and the result with the signature was returned to the client.
3. Submit: The client verified that the number of signatures from the organizations satisfied the endorsement policy. If it was satisfied, the transaction was sent to the ordering service, which ordered the series of transactions in chronological order and created the block of the transactions.
4. Broadcast: The block was delivered to all nodes.
5. Commit: If each block was validated to fulfill the endorsement policy and was to be well formed, the block was appended to the chain in each node.

All data, including the client hashchain, were registered in the blockchain network via relay servers to secure the tamper-resistance of the data. In contrast, the secure string for the calculation of the hash value was preserved in the client device. At the end of the study, the secure string was sent to the blockchain network to verify the hashchain. The client hash values were calculated on the mobile phone based on medical data, the secure string, and the previous hash value using the SHA-256 algorithm [39].

### Test Scenarios

There are issues regarding cybersecurity (ie, concerning external actors) and governance/authenticity (eg, internal actors like researchers) in medical data management. The tamper-resistance of the data registered in the blockchain network against external attack has been proven in previous studies. The reliability of the data against internal actors in the blockchain network can also be guaranteed by managing the accounts for each node by different stakeholders, such as pharmaceutical companies, contract research organizations, and regulatory agencies. Here, we evaluated how the data manipulation before registration to the blockchain network can be detected and distinguished by simulating the following malicious access. The artificial data were created for each scenario and tested if the fraudulent access were detected and the original data can be distinguished from the illegal data. Since the results of the manipulation of the data are deterministic due to collision-resistant hash functions of SHA-256 [42], we verified the result of a single manipulation in each scenario. Since the accounts for nodes of the blockchain network and the relay servers were managed by independent departments, both internal and external actors can be simulated by hacking each server.

1. Attack on the relay server: To simulate an artificial attack on the relay server (ie, outside actors) or misconduct by the owner of the relay server (ie, inside actors) during the clinical trial, one of the relay servers was hacked. The data sent from a client device can be modified before registration to the blockchain network by the malicious access. In this case, the secure string for the calculation of a hash value in the client device was not stolen. Hence, the client hash value was calculated with the previous hash value, modified medical data, and the incorrect string.
2. Attack on the authentication server: To simulate an artificial attack on the authentication server (ie, outside actors) or misconduct by the owner of the authentication server (ie, inside actors) during the clinical trial, the authentication key of an existing account on the authentication server was stolen and the data of the hacked account was uploaded by multiple devices. In this case, the secure string for the calculation of a hash value in the client device was not stolen. Hence, the client hash value was calculated with the previous hash value, modified medical data, and the incorrect string.
3. Attack on the client device: The secure string preserved in the client device was stolen by an attacker using a mobile malware root exploit. The authentication information to the relay servers was also stolen by the malicious infection. Hence, the client hash value was calculated by different devices with the previous hash value, modified medical data, and the correct secure string.

## Results

### Design of the Client for Blockchain Network in mHealth

**Figure 1:**
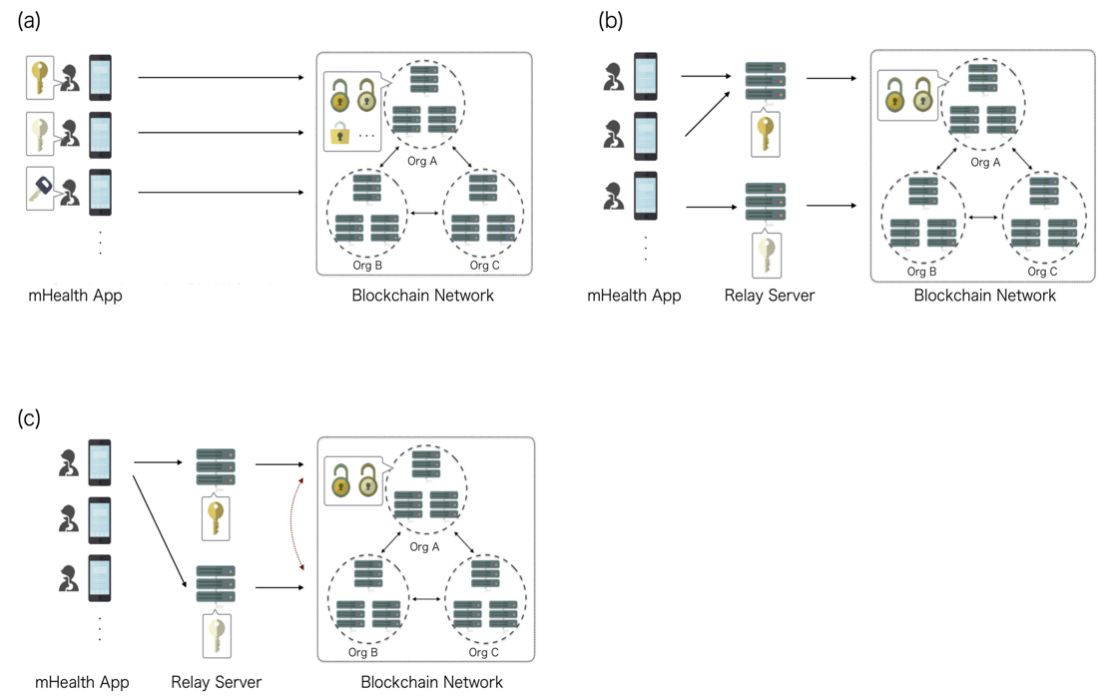
mHealth system architecture, which sends the medical data to the blockchain network. Data sent to (a) the blockchain network without relay servers; client devices are dealt with as nodes of the blockchain network so that mHealth app needs to contain software development kits for blockchain; (b) the blockchain via a single relay server; public keys for each relay server should be managed; (c) the blockchain via multiple relay servers; public keys for each relay server should be managed. Data reliability can be verified by comparing the data to be registered (red line).

In our previous study, the mHealth data obtained by a mobile phone were uploaded to the blockchain network. We then evaluated the robustness of the network and the tamper-resistance of the data in the blockchain network [24]. To advance further into the practical usage of blockchain in mHealth, it is necessary to design how the client devices, such as mobile phones, send medical data to the blockchain network. Client devices can send data to the blockchain network directly, but with this architecture, the mHealth app that patients installed to their mobile phone needs to have SDKs for blockchain. The blockchain network will also receive access from unspecified clients due to a lack of IP address restriction. In that situation, there are risks that unspecified clients can store medical information in the blockchain network using SDKs to read the data. The system is resistant against data tampering, but the operational costs increase since they must manage private keys for each client device (Figure 1). In order to overcome these obstacles, the usage of relay servers is one possible option. With the architecture using relay servers, the blockchain network can restrict the access by IP address selection and it is not necessary for the mHealth app to include SDKs for blockchain. With this architecture, blockchain networks were protected against unspecified access from the internet and the functions of SDKs in the relay server can be predetermined as write-only, resulting in the protection of the medical data stored in the blockchain network. On the other hand, there are risks that the hacking of the relay server will result in impersonation (Figure 1). To balance these trade-offs, we propose the following architecture of the client for the blockchain network in mHealth. The client devices, such as mobile phones, send their data to multiple relay servers and the data are compared between relay servers and verified. After verification, the relay server, which has permission to access by IP address restriction, sends the data to the blockchain network using write-only functions of SDKs. With this system, medical data stored in the blockchain network are protected against access from the internet and are resistant against the risks of server hacking (Figure 1).

### Authentication Between Clients and Relay Servers

**Figure 2:**
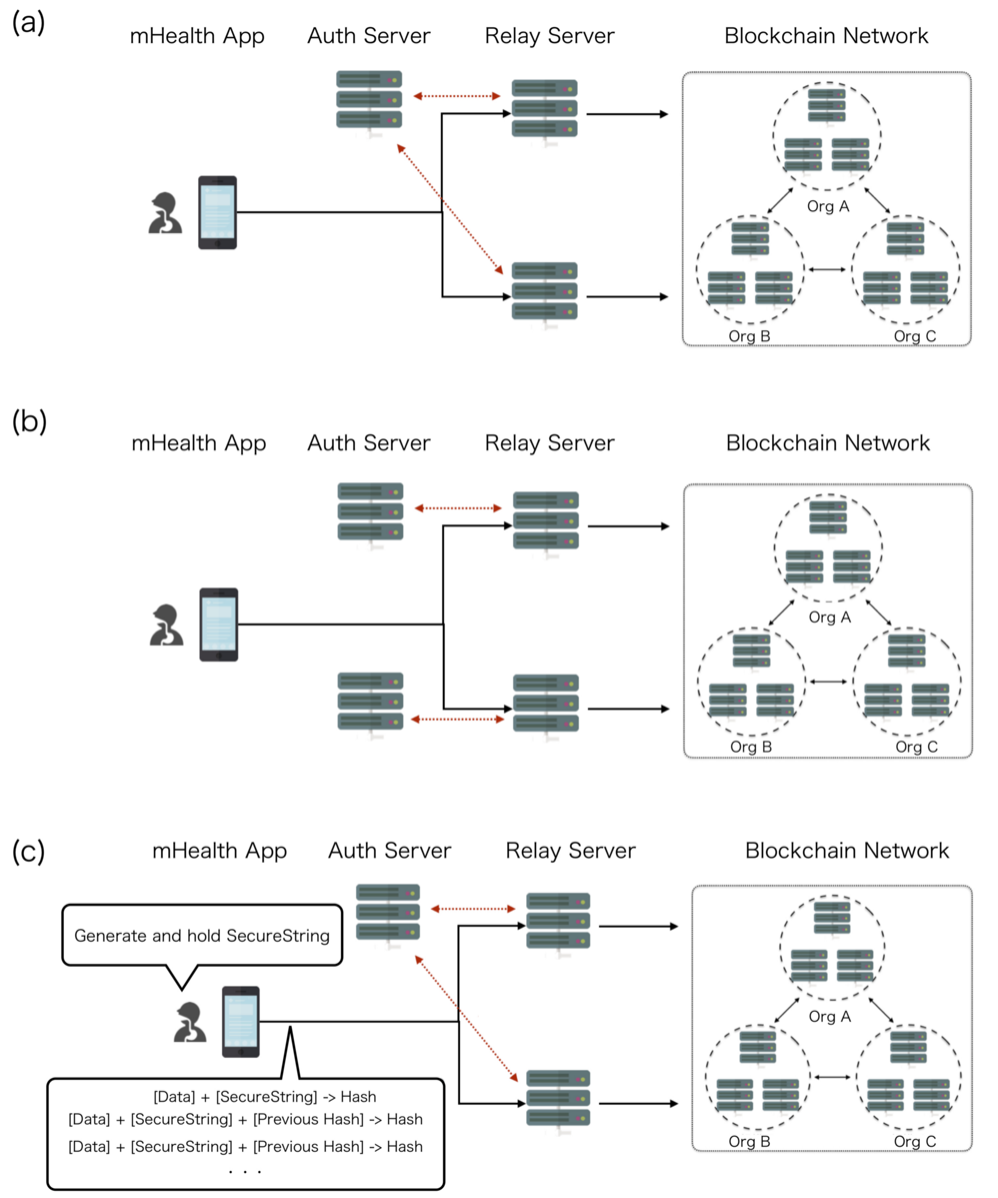
Authentication of client devices and relay servers. Client devices authenticated by (a) a single common authentication server; the system is vulnerable against server hacking; (b) multiple authentication servers for each relay servers; (c) a single common authentication server. In addition to authentication, client devices calculate a hash value based on data, secure string, and previous hash value, so the hash value consists of the chain structure (client hashchain).

For the usage of relay servers described above, it is indispensable to carefully design the authentication of client devices to send data to relay servers. It is possible that a single common server gives an authentication to client devices, but with this architecture, the system is vulnerable to server hacking. Malicious access to the single authentication server can result in impersonation (Figure 2). The alternative is to set an authentication server for each relay server. Although the risks of impersonation by server hacking can be reduced, the operational costs for authentication increase. In addition, it is not possible to verify the reliability of the original data if multiple authentication servers were maliciously accessed (Figure 2). In order to solve these problems, we implemented a single common authentication server with another method, using a hash value calculated on the client devices. As an initial setting, the client device generates and preserves a secure string. The client device calculates a hash value based on the medical data and the secure string, as well as the previous hash value using the SHA-256 hash algorithm. Thus, the hash value comprises the chain structure. The hash value was also registered in the blockchain network along with the medical data in order to guarantee tamper-resistance of the value, although the secure string was preserved in the client device. It is possible to verify the reliability of data using the secure string preserved in the client devices and to retrospectively reject impersonation after finishing the clinical trials. Even when a relay server was hacked by malicious access, we can verify the correct data based on the client hashchain and the secure string preserved in the client device. Since the hash value calculated in the client device makes up the chain structure, we called the technique “client hashchain” in contrast to blockchain (Figure 2). In the case of the device having been destroyed or disabled prior to the conclusion of the study, the secure string could be sent beforehand to the user’s personal storage, such as their email box.

### Application of the Proposed System to mHealth and Data Management in a Clinical Trial

In order to validate the system proposed above, we have implemented the architecture into the mHealth app. The app was designed to treat insomnia patients based on CBTi and collects medical data using mobile phones. The app generates a secure string at login and stores it on the client device. The app also calculates a hash value based on the medical data, the previous hash value, and the secure string so that the hash values make up the chain structure. The medical data collected with the app, as well as the hash value, were sent to the blockchain network via relay servers. We used three relay servers and the app selected two relay servers at random to send the data to the blockchain network. The blockchain network comprised three organizations, which contain two validating peers.

**Figure 3:**
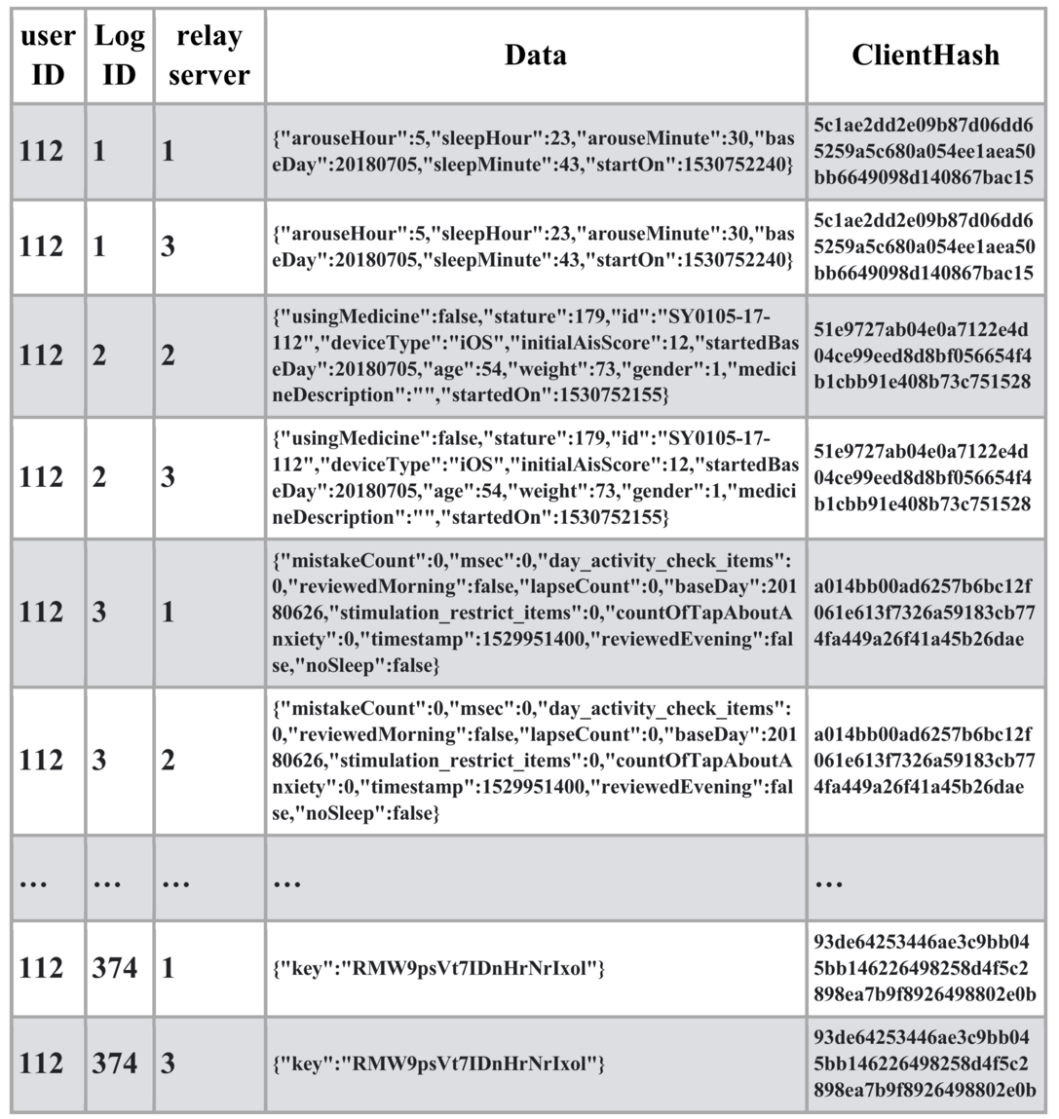
User data along with client hashchain registered to blockchain network.

With these systems, we conducted the clinical trial on the mHealth app for insomnia patients. Informed consent was obtained from the patients and the app account was provided by the medical doctor. mHealth data were collected with the mobile app and sent to the blockchain network via relay servers along with the client hash value (Figure 3). In the client devices, client hash values were calculated based on mHealth data, the previous client hash value, and the secure string stored on the client device. The client hash value constitutes the chain structure and proves the origin of the sequential data.

Both mHealth data and the client hash value were sent to the blockchain network to be registered in the ledger. The ledger is made up of the blockchain, sequenced records in blocks, and a state database. Each node of the blockchain maintains a copy of the ledger. If the transaction was validated under the endorsement policy, the chaincode was executed and the block of the transaction was appended in each node. The mHealth data and the client hash value were stored in CouchDB and the blockchain. The block includes a hash of the block’s transactions, as well as a hash of the prior block. In this way, it is not possible to tamper with the ledger data without breaking the hash links.

Although the block size is limited in blockchain, it is enough for our medical data since the clinical indicators are stored as JSON data. In addition, the transaction throughput in the Hyperledger Fabric platform that we used is 2250 transactions per second [27]. In contrast, in a permissionless network or public network, such as Bitcoin and Ethereum, it takes 600 seconds and 10 seconds respectively to write a transaction on the ledger [28]. Since the number of transactions in our mHealth system occur several times per day for every patient, the transaction performance of the blockchain will not be a bottleneck.

### Validation of the Resistance Against Attack on the Relay Server

To investigate the resistance of our proposed system, we first simulated an artificial attack on the relay server (ie, outside actors) or misconduct by the owner of the relay server (ie, inside actors) and evaluated if the malicious access was detected and if the original data are distinguishable from the illegal data. As described above, the client device sends the data to the blockchain network via multiple relay servers. We used three relay servers and the app selected two relay servers at random to send the data to the blockchain network. If one of the relay servers is hacked, the attacker can modify the data sent from the client device prior to blockchain submission and steal the authorization token. In this case, the secure string used in the calculation of the client hashchain was not stolen by the attacker.

**Figure 4:**
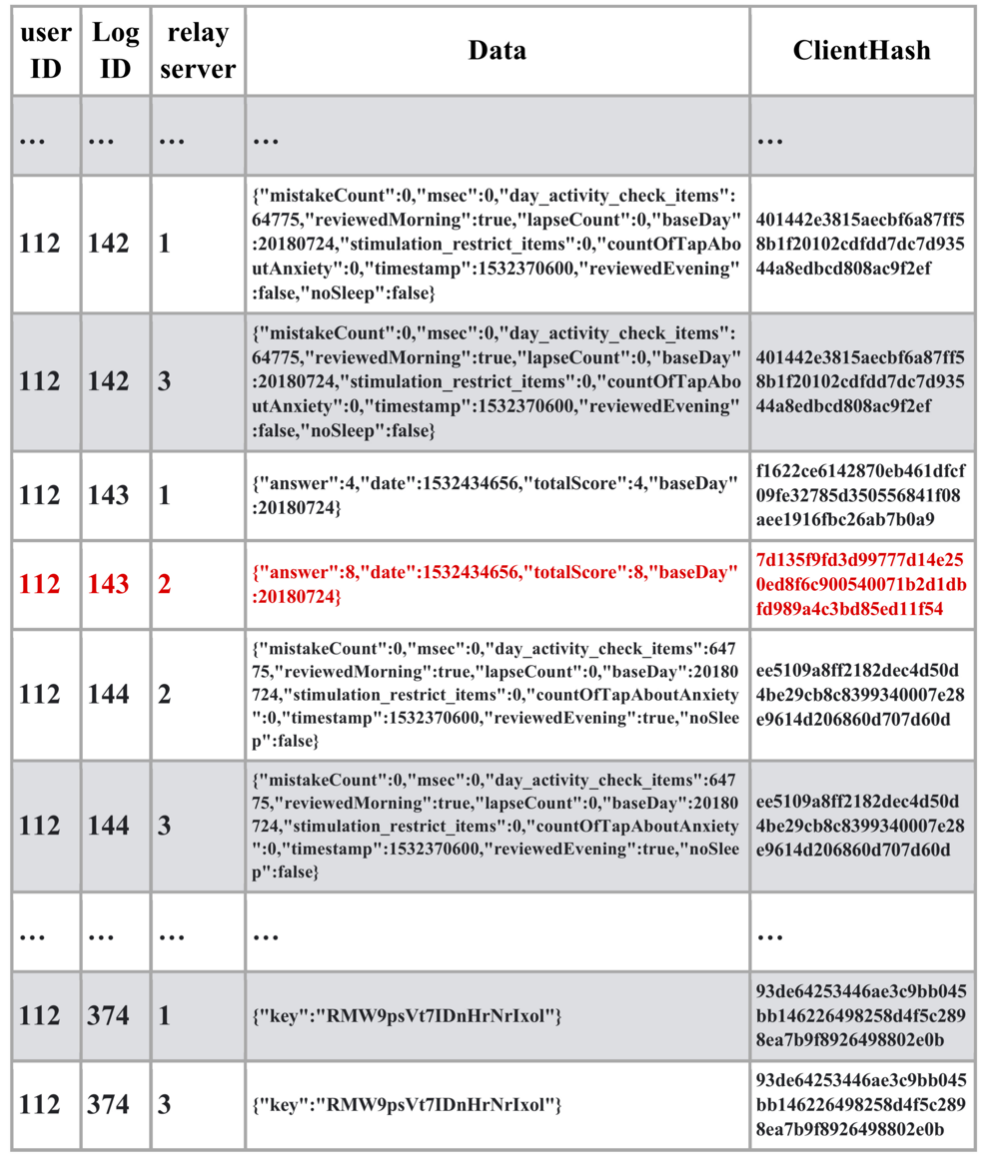
Relay server hacked and data modified by access fraud (client hash value in red).

As shown in Figure 4, an attacker hacked relay server #2 and modified some medical data as well as the client hash value. In this case, the client hash value was calculated with the previous hash value, modified medical data, and the incorrect string. The access fraud can be detected by the mismatch between the data sent from other relay servers. It is also possible to distinguish and reject illegal data from original data automatically using the client hashchain. At the end of the clinical trial, the device sent the secure string to the blockchain to make it possible to validate the uploaded data retrospectively. Since all the medical data and the client hash values were stored in the blockchain network, it is not possible to rewrite the hash value based on the secure string, which was sent via the relay servers. Legal data can be guaranteed by combining the client hashchain, which rejects impersonation, with the blockchain, which provides tamper-proof history.

### Validation of the Resistance Against Attack on the Authentication Server

We next simulated an artificial attack on the authentication server (ie, outside actors) or misconduct by the owner of the authentication server (ie, inside actors) during the clinical trial and evaluated whether the malicious access was detected if the original data are distinguishable from the illegal data. The authentication server possesses the authentication keys of every account. If the authentication key was stolen, the attacker can send illegal data from different devices. In this case, the secure string used in the calculation of the client hashchain was not stolen by the attacker.

**Figure 5:**
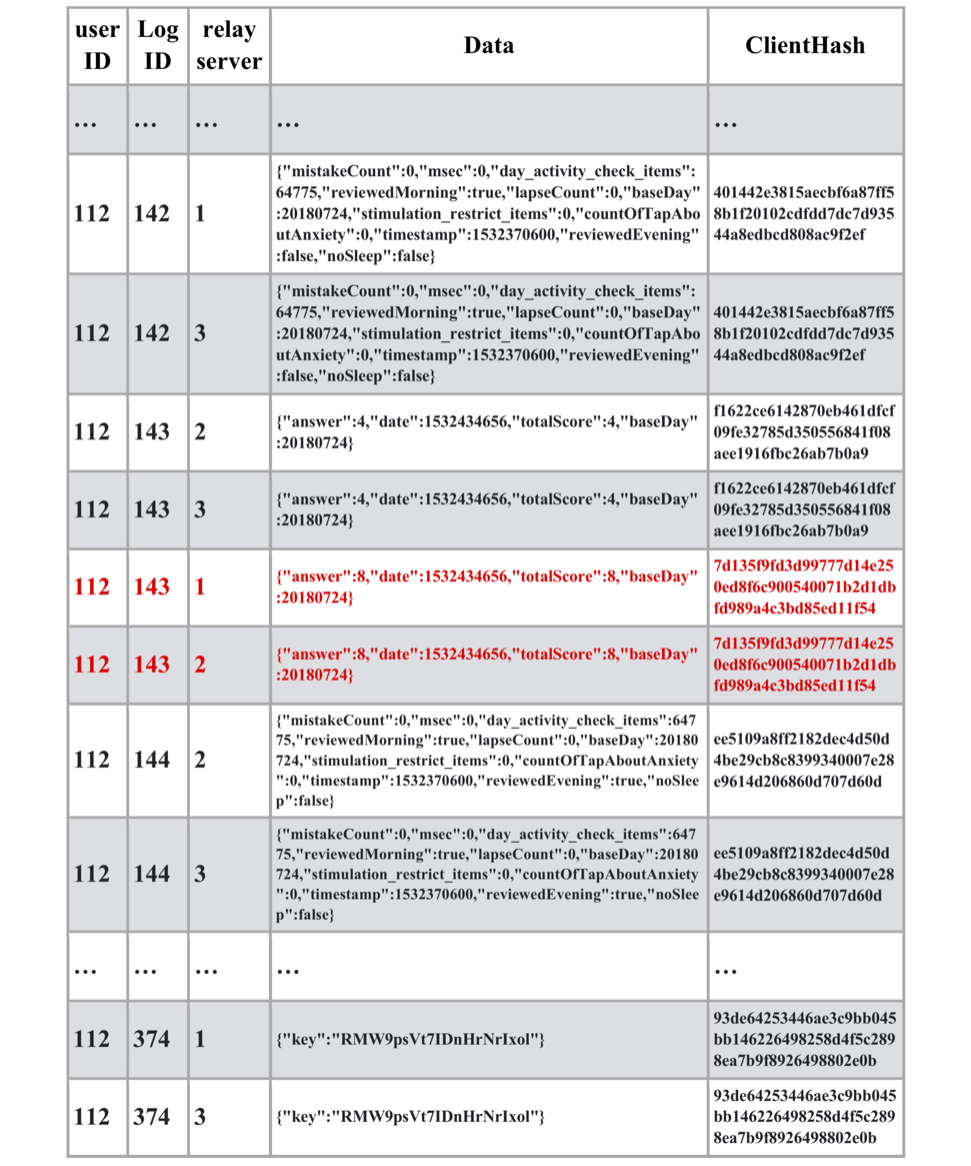
Authentication server hacked and data generated from multiple devices using the authentication key (authentication key was stolen and the attacker created illegal data from different devices in red).

As shown in Figure 5, using the stolen authentication key, multiple data with the same log ID were generated and sent to the blockchain network via relay servers by the attacker. Fraud detection can be achieved by identifying branched data. Furthermore, it is also possible to distinguish and reject illegal data from original data automatically using the client hashchain. At the end of the clinical trial, the device sent the secure string to the blockchain to enable the validation of the uploaded data retrospectively. Since all medical data and the client hash values were stored in the blockchain network, it is not possible to rewrite the hash value based on the secure string that was sent via relay servers.

### Validation of the Resistance Against Attack on the Client Device

To further investigate the resistance of our proposed system, we next simulated an artificial attack on the client device and evaluated if the malicious access was detected and if the original data are distinguishable from the illegal data. One of the most dangerous attacks is the malware root exploit, which enables the attacker to obtain the victim’s private key. In this case, the authentication key as well as the secure string were stolen by the attacker, resulting in a more serious situation.

**Figure 6:**
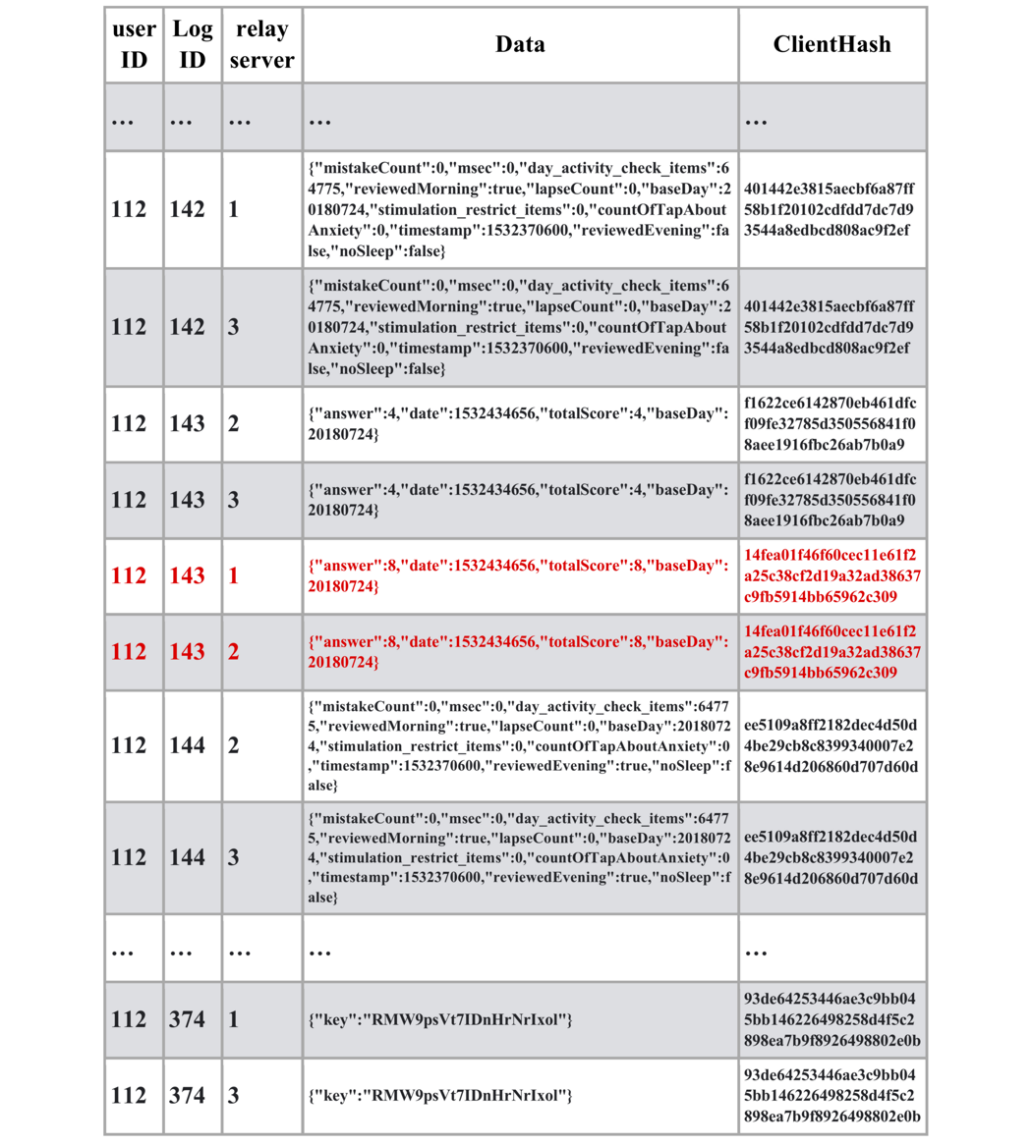
Client device washacked by malware root exploit and data generated from multiple devices using the authentication key (authentication key as well as the secure string for the client hash value were stolen by root exploit and the attacker created illegal data from different device in red).

As shown in Figure 6, using the stolen authentication key, multiple data with the same log ID were generated and sent to the blockchain network. Fraud detection can be achieved by identifying the branched data. However, it is not possible to distinguish illegal data from original data automatically using the client hashchain because the attacker has stolen the secure string to calculate the client hash value. In this case, however, it is possible to judge which are the original data by checking the data in the patient’s device offline, based on the fraud detection.

## Discussion

### Principal Findings

In this study, we have developed a secure and scalable mHealth system using relay servers and blockchain combined with a client hashchain. Although blockchain technology provides tamper-resistance to medical data [24], the security was limited to the registered data and it cannot distinguish between original data and impersonated data. In addition, scalability will be compromised if the client devices were dealt with as a node of blockchain network. With our proposed system, we have shown that these problems can be resolved.

Bitcoin was the first implementation of blockchain as a digital asset in widespread use. Although bitcoin can be used as a platform for preventing data tampering, it is not appropriate since it is an open network and massive computing power is necessary for proof of work (PoW) to obtain consensus [43]. Private blockchain networks, such as Hyperledger Fabric, are more appropriate for the management of medical data since the node of stakeholders can be controlled. In addition, it is possible to process more transactions in a private blockchain without PoW. Using a private blockchain network, we used relay servers to send the data from authorized clients. With this architecture, it is not necessary to incorporate SDKs on client devices. To make the system robust against hacking of the relay server, the app sent data to the blockchain network via multiple relay servers. Even when one of the relay servers was hacked, we could detect access fraud and distinguish the original data from modified data. In our study, we used three relay servers and two were randomly selected to send the data. The robustness of the system against server attack can be increased if we use more relay servers, for instance, if three out of five relay servers are randomly selected to send the data.

To further clarify the origin of the data, we combined the client hashchain with the private blockchain. Hash values combined with the blockchain have been used as the metadata in a previous study for the management of rights for digital contents [44]. In contrast, we used hash values calculated on the client device to protect against impersonation and verify the origin of the data by chaining them. Hash values with chain structure (client hashchain) enable the identification of the original data sent from a specific client, which stores the secure string. In addition, in combination with the blockchain, the system also ensured tamper-resistance and the reliability of the hash values to prevent impersonation. We have shown that fraudulent data by compromised relay servers can be detected and distinguished from original data using the client hashchain. Even when the secure string used in calculating the client hash value was stolen by the attacker with root exploit [45], it is possible to detect the malformation in the branching of the chaincode. Based on the detection of the malformation, the researchers can ask the patients and check which are the original data. Therefore, the system is highly resistant against impersonation and tampering.

In this study, we designed the architecture for mHealth and verified the performance in a clinical study. Although mHealth is suitable for collecting medical data, such as patient reported outcomes [46], by changing the client device from patients’ mobile phone to computers in medical institutions, the system can also be applied to clinical trials that use electronic data capture [47]. In addition, we could deploy smart contract, which is called chaincode in Hyperledger Fabric, to each node of the blockchain network to execute transactions. Since the smart contract may have the function to transform medical data into the determined format, it is possible to automatically complete the case report form of each patient if the app has access to additional medical data by deploying the smart contract for the clinical trial [25,48].

The system enables the verification of the accuracy of the medical data without confirmation by the third party, such as a contracted research organization, so that it is possible to reduce the cost of clinical trials as well as the possibility of human error. Thus, our system based on the blockchain technology combined with a client hashchain may enhance the development of drugs and medical devices.

### Limitations

Further studies are needed to verify the scalability of the system for conducting multiple clinical trials simultaneously. In Hyperledger Fabric v1.0, it is possible to partition the network and define a communication channel using an ordering service, which enables multiple clinical trials to be conducted in the same system [49]. Although the transaction throughput in the Hyperledger Fabric platform that we used is much higher than public blockchain, one drawback of the private network described here is preventing 51% of attacks in networks that are composed of a limited number of nodes without public validation.

Although our system is resistant against impersonation and tampering, hacking of the client device is a great threat. Root exploit is a type of malware attackers use to modify the Android operating system kernel such that attackers are able to gain super-user privileges. When attackers gain root of the operating system kernel, they also gain access to full administrator privileges. Through this, attackers are able to install other malware types, such as botnets, worms, or Trojans into the system. Further studies like root exploit detection [45] may be beneficial for the improved security of the system.

### Conclusion

In this study, we designed a secure and scalable mHealth system using blockchain. A client hashchain was combined with the blockchain network to protect against impersonation, enabling the usage of relay servers and reducing the complexity of authentication of client devices for mHealth. The system was validated in the clinical trial, and the resistance against various fraud attacks was evaluated.

## Abbreviations

CBTi: cognitive behavioral therapy for insomnia
IoT: internet of things
IP: Internet Protocol
JSON: JavaScript Object Notation
PoW: proof of work
SDK: software development kit
SHA: secure hash algorithm
